# Invasive Fungal Disease in Patients with Chronic Lymphocytic Leukemia in Japan: A Retrospective Database Study

**DOI:** 10.3390/curroncol29050264

**Published:** 2022-05-04

**Authors:** Takeo Yasu, Kotono Sakurai, Manabu Akazawa

**Affiliations:** 1 Pharmaceutical Education and Research Center, Department of Medicinal Therapy Research, Meiji Pharmaceutical University, Noshio, Kiyose 204-8588, Japan; y171136@std.my-pharm.ac.jp; 2 Department of Public Health and Epidemiology, Meiji Pharmaceutical University, Noshio, Kiyose 204-8588, Japan; makazawa@my-pharm.ac.jp

**Keywords:** chronic lymphocytic leukemia, invasive fungal disease, claim database

## Abstract

Invasive fungal disease (IFD) is an important cause of morbidity and mortality in patients with hematological malignancies. As chronic lymphocytic leukemia (CLL) is a rare hematological malignancy in Japan, IFD incidence in Japanese patients with CLL is unclear. This study aimed to investigate IFD incidence in Japanese patients with CLL. This retrospective cohort study used data of patients with CLL registered between April 2008 and December 2019 in the Medical Data Vision database (*n* = 3484). IFD incidence after CLL diagnosis in the watch-and-wait (WW) and drug therapy (DT) groups was 1.5% and 9.2%, respectively. The most common type of IFD was invasive aspergillosis (28.1%). Cox proportional hazards multivariate analysis revealed that DT (hazard ratio [HR]: 2.13) and steroid use (HR: 4.19) were significantly associated with IFD occurrence. IFD incidence was significantly higher in the DT group than in the WW group (log-rank *p* < 0.001); however, there was no significant between-group difference in the time to IFD onset or the type of IFD (*p* = 0.09). This study determined the incidence of IFD in patients with CLL during WW. Physicians should monitor for IFD, even among patients with CLL undergoing the WW protocol.

## 1. Introduction

Invasive fungal disease (IFD) is an infection associated with high mortality, ranging from 29–90% [1,2]. Thus, it is important to identify trends in the incidence of IFD because delays in treatment initiation can lead to even higher mortality rates. Hematological malignancy is one of the strongest predictors of IFD-associated mortality [1]. In a retrospective observational study in Australia, 1.33% of patients with chronic lymphocytic leukemia (CLL) developed IFD within 12 months of cancer diagnosis [3]. Although clinical outcomes for patients with hematologic malignancies have improved in recent years, evidence on the epidemiology of IFD in Asia is still limited, with only a few reports [4,5,6]. CLL is a tumor of atypical B lymphocytes that accumulate in the bone marrow, peripheral blood, and lymphoid tissues. CLL is relatively common in Europe and the United States, accounting for approximately 25% of all hematopoietic malignancies. In contrast, CLL is a rare hematological malignancy in Japan, with an incidence of only 1–2% [7,8]. Therefore, the incidence of IFD in Japanese patients with CLL remains unclear. Understanding the epidemiology of IFD in Japan can potentially guide further antifungal strategies.

CLL is an indolent B cell leukemia that often occurs in older individuals. Therefore, the characteristic treatment strategy for CLL in the early stages, when the patient does not have symptoms, is the watch-and-wait (WW), which is also an important approach without drug therapy (DT) [9]. Although hematologic malignancies with or without neutropenia are risk factors for IFD [10], no studies have evaluated IFD surveillance in patients diagnosed with CLL by distinguishing between WW patients without neutropenia and those receiving DT. In addition, new targeted therapies have been initiated for patients with CLL, such as ibrutinib [11], an irreversible inhibitor of Bruton’s tyrosine kinase (BTK). Several reports have recently shown that ibrutinib use is associated with IFD [12,13,14]. Thus, this study aimed to assess the incidence of IFD and its risk factors in patients with CLL who undergo the WW protocol, and those who receive DT, using data from Japanese health insurance claims.

## 2. Materials and Methods

### 2.1. Study Design and Patients

This retrospective cohort study was conducted using data from the large-scale Medical Data Vision (MDV) database [15], a nationwide hospital-based claims database that covers patients who use hospitals with the diagnostic procedure combination (DPC) payment system in Japan. All patient data are encrypted prior to database entry. The MDV database contains information such as disease code (International Classification of Diseases-10 (ICD-10)), prescription details, and medical treatment, in addition to patients’ basic information.

A total of 258,565 patients with hematological malignancies, identified using ICD-10 codes C81-C96 and D46, were registered in the database between April 2008 and December 2019. The patients who met the following inclusion criteria were evaluated: (1) ICD-10 code C911, (2) age ≥16 years, and (3) ICD-10 code C911 after April 2008. Differences in the incidence and risk factors of IFD between patients who underwent the WW protocol therapy and those who received DT were evaluated. The DT group included patients who received at least one prescription for drugs for CLL (fludarabine, cyclophosphamide, bendamustine, rituximab, ofatumumab, alemtuzumab, or ibrutinib). Patients who did not receive any prescription for anticancer drugs after diagnosis were categorized into the WW group.

### 2.2. Data Collection and Variable Definition

Patient data were collected from the start of observation, defined as the month of CLL diagnosis. Patients with CLL who did not develop IFD were observed until the end of the study period. If a patient developed IFD at a particular time point during the study period, observation was terminated at that time point. IFD onset was defined as the first day by which the patient (1) was assigned an ICD-10 code for IFD (B371, B375-8, B420, B427-9, B430-2, B438-9, B440-2, B447-9, B450-3, B457-9, B460-5, B468-9, B470-1, B479, B480-4, B487-8, B49, or B59) and (2) was prescribed at least one intravenous antifungal (i.e., fluconazole, fosfluconazole, itraconazole, voriconazole, micafungin, caspofungin, liposomal amphotericin b, or trimethoprim (TMP)-sulfamethoxazole) for IFD or 9–12 tablets of trimethoprim-sulfamethoxazole daily during hospitalization. The Infectious Diseases Society of America guidelines recommend intravenous preparations for the initial treatment of IFD [16]; however, because the treatment of pneumocystis pneumonia (PCP) involves intravenous or oral administration of TMP-sulfamethoxazole 15–20 mg/kg daily (doses based on TMP), we also included the prescription of 9–12 TMP-sulfamethoxazole tablets daily as treatment for IFD [17].

The Charlson Comorbidity Index (CCI) score was assessed using the ICD-10 code as reported by Quan et al. [18]. CCI scores were derived from the following items: myocardial infarction, congestive heart failure, peripheral vascular disease, cerebrovascular disease, dementia, chronic pulmonary disease, rheumatic disease, peptic ulcer disease, mild liver disease, diabetes without chronic complications, hemiplegia or paraplegia, renal disease, diabetes with chronic complications, solid tumor, malignancy, moderate or severe liver disease, metastatic solid tumor, and acquired immunodeficiency syndrome/human immunodeficiency virus. We also collected information on diabetes mellitus and steroid drugs use as risk factors for IFD [19]. Diabetes mellitus was defined by ICD-10 codes (E10x, E11x, E12x, E13x, or E14x), and steroid use was defined as at least one prescription for corticosteroid drugs.

### 2.3. Statistical Analysis

Among the characteristics of patients and the duration of the onset of IFD between the WW and DT groups, continuous variables are expressed as median (interquartile range (IQR)) and were analyzed using the Mann–Whitney U test; categorical variables are expressed as absolute numbers or percentages and were analyzed using Fisher’s exact test. The differences in characteristics of IFD in the two groups were analyzed using Fisher’s exact test. The cumulative incidence of IFD after CLL diagnosis was compared between the two groups using the log-rank test; a Cox proportional hazards regression model was used to assess risk factors for developing IFD. Univariate and multivariate analyses were performed for the explanatory variables sex, age at baseline (≥75 years), CCI score (≥5), diabetes, steroid use, and DT, and their hazard ratios (HRs) were calculated. All statistical analyses were performed using EZR (Saitama Medical Center, Jichi Medical University, Saitama, Japan) [20]. Statistical significance was set at *p*-values < 0.05. The impact on IFD for each anticancer drug was evaluated by the incidence of IFD in patients for whom each anticancer drug was prescribed at least once.

## 3. Results

### 3.1. Patient Characteristics

**Figure 1:**
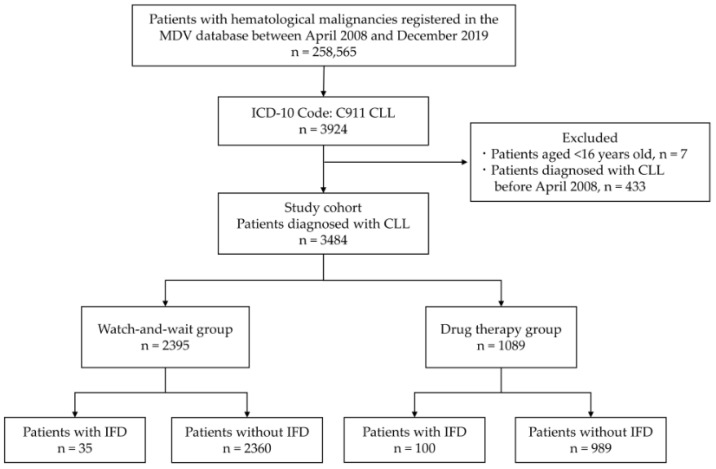
Patient selection flow diagram. Abbreviations: MDV, Medical Data Vision; CLL, chronic lymphocytic leukemia; IFD, invasive fungal disease; ICD-10, International Classification of Diseases 10th edition.

In total, 3484 patients with CLL were included in this study (Figure 1). The median age at cohort entry was 72 years (IQR, 64–80 years), and 2034 patients (58.4%) were male. The median CCI score was 1 (IQR, 1–2), and the median follow-up duration was 851 days (IQR, 221–1788 days). Overall, 2395 and 1089 patients belonged to the WW and DT groups, respectively. The WW group included a significantly higher number of patients who were older, male, used steroids, and had a CCI score ≥ 5. Among the group of treated patients, cyclophosphamide was the most common anticancer drug (47.0%), followed by rituximab (45.0%), fludarabine (39.3%), and ibrutinib (21.2%). The patient characteristics by group are summarized in Table 1.

### 3.2. Incidence and Risk Factors of IFD

**Figure 2:**
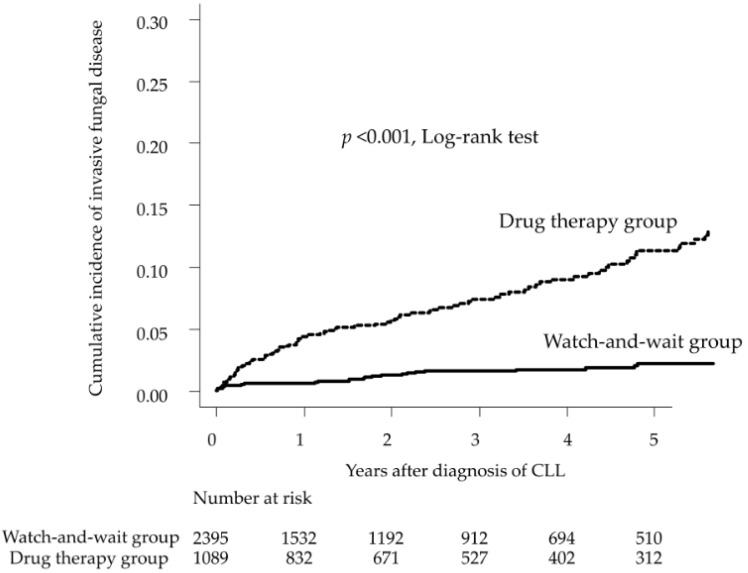
Cumulative incidence of invasive fungal disease after chronic lymphocytic leukemia diagnosis.

IFD occurred in 135 (3.9%), 35 (1.5%), and 100 (9.2%) patients in the overall, WW, and DT groups, respectively. The 1- and 5-year cumulative incidence rates of IFD were 0.6% and 2.3%, respectively, in the WW group and 4.5% and 11.3%, respectively, in the DT group (log-rank *p* < 0.001) (Figure 2). Invasive aspergillosis was the most common IFD (*n* = 38 patients, 28.1%), followed by unspecified IFD, other IFD not otherwise specified (NOS), and PCP. There were nine patients (6.7%) with invasive candidiasis, all of whom belonged to the DT group. There was one case each of cryptococcosis in the WW and DT groups. None of the IFDs were significantly different between the groups (Table 2).

**Figure 3:**
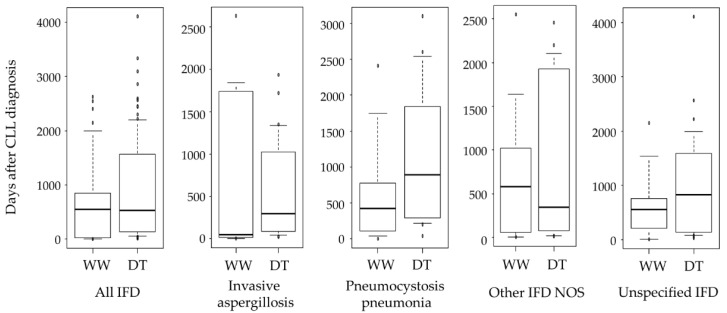
Duration of the onset of invasive fungal diseases after diagnosis of chronic lymphocytic leukemia. CLL, chronic lymphocytic leukemia; WW, watch-and-wait group; DT, drug therapy group; IFD, invasive fungal disease; NOS, not otherwise specified.

In the Cox proportional hazards’ multivariate analysis, steroid use (HR: 2.13, 95% confidence interval [CI]: 1.50–3.30, *p* < 0.001) and DT (HR: 4.19, 95% CI: 2.82–6.23, *p* < 0.001) were significantly associated with the occurrence of IFD (Table 3). The incidence of IFD in patients who received alemtuzumab was 38.5%, the highest of any anticancer agent used to treat CLL. In contrast, the incidence of IFD in patients who received ibrutinib was the lowest at 8.2% (Table 4). One case of cryptococcosis was observed in a patient who received fludarabine; 38.5% of patients who received alemtuzumab developed invasive aspergillosis. The median time from CLL diagnosis to IFD onset was 551 days (IQR, 104–1445 days). There was no difference in the median time from CLL diagnosis to IFD onset for all IFDs (Mann–Whitney U test, *p* = 0.09) (Figure 3). Disease duration was the shortest for invasive aspergillosis in the WW (45.4 days) and DT (297.5 days) groups.

## 4. Discussion

Data on IFD in Japanese patients with CLL are scarce owing to the rarity of this malignancy, but IFD is an important cause of morbidity and mortality. In this study of IFD in CLL patients in Japan, *Aspergillus* was the most common causative fungus in this study, accounting for 28.1% of all IFDs. The incidence of IFD was significantly higher in the DT group than in the WW group, but there was no significant between-group difference in the median time to IFD onset after CLL diagnosis (*p* = 0.09).

CLL is a rare hematologic disease in Japan; thus, we determined the incidence of IFD in Japanese patients with CLL using the claims database. The incidence of IFD was higher in this study than that in the study by Valentine et al. (1.33%) [3]. However, when the period covered was set to 12 months after the diagnosis of CLL, as in Valentine et al.’s study [3], the incidence of IFD was similar at 1.81% (*n* = 63). Thus, the difference in the incidence of IFD in patients with CLL among Japan, Europe, and the United States was small. Chemotherapy-induced neutropenia is a risk factor for IFD [10]. Consistently, the incidence of IFD in the DT group was significantly higher than that in the WW group in the current study. However, there was no significant difference in the median time to IFD onset between the WW and DT groups. Interestingly, the median time to onset of invasive aspergillosis after CLL diagnosis was earlier at 45.4 days in the WW group. Age has been reported as a risk factor for the development of invasive aspergillosis [21]. The WW group was older than the DT group (*p* = 0.036), suggesting that age may be a factor in the early development of invasive aspergillosis. In addition, although prophylactic administration of antifungal agents was not considered in this study, the presence or absence of prophylactic administration given in the DT group and generally not given in the WW group may be a factor in the early onset of invasive aspergillosis. Given that CLL has few subjective symptoms in its early stages, there are patients in whom CLL was diagnosed only at IFD onset. Four such cases were identified in this study.

Steroid use significantly increased the risk of IFD, consistent with findings of a previous study [19]. CLL may present with autoimmune diseases, such as autoimmune hemolytic anemia and autoimmune thrombocytopenia, and, thus, patients with CLL are often prescribed steroids to treat autoimmune diseases. Steroids are risk factors for IFD because they decrease cellular immunity [10]. As such, patients taking steroids should be carefully monitored, regardless of DT for CLL. However, steroid use was significantly more common in the DT group as an antiemetic agent. Steroid use, excluding use to prevent chemotherapy-induced nausea and vomiting, was not a risk for developing IFD (HR: 1.58, 95% CI: 0.94–2.66, *p* = 0.09). Therefore, the results relating steroid use to the development of IFD may be confounding. In 2014, 42.2% of 2000 Japanese patients with CLL underwent the WW approach [22]. The proportion increased to 68.7% in this study. As this study included data up to 2019, the aging of the target patients may have influenced the increase in the number of patients undergoing the WW protocol. Patients who were older and had a CCI score of ≥5 were significantly more likely to be in the WW group because of the difficulty in performing DT. By classifying patients with CLL into the WW and DT groups, we could clarify the incidence and favorable timing of IFD treatment in WW patients, which is expected to increase in an aging society.

Among causative fungal agents of IFDs, invasive *Aspergillus* was the predominant species (28.1%), concordant with the findings of recent studies [23,24]. In Japan, *Aspergillus* and *Candida* are the most frequent organisms causing IFDs [3,4]. Our results also showed that invasive aspergillosis is the most common IFD. Invasive *Candida* was observed only in the DT group, possibly because catheter-related bloodstream infections via medical devices, such as central venous catheters, and DT are risk factors [25]. We did not assess the incidence of IFD according to the DT regimen; however, the results showed that patients treated with alemtuzumab had the highest incidence of IFD among anticancer agents for CLL treatment. Alemtuzumab has been previously reported as a risk factor for IFD because it suppresses T-cell immunity [26], and consistent findings were obtained in this study.

The combination of fludarabine, cyclophosphamide, and rituximab is the standard modality for treatment-naïve young and fit patients with CLL [27], and fludarabine is a key drug for CLL treatment. Fludarabine carries a long-term risk of IFD because it continuously decreases the CD4 count for several months even after treatment discontinuation [26]. Alemtuzumab, a humanized anti-CD52 monoclonal antibody, has therapeutic effects on untreated and fludarabine-refractory CLL. However, alemtuzumab acts not only on CLL cells but also on normal immune cells, and the cumulative effect can adversely affect a patient’s protective response to fungal infection [28]. Similar to fludarabine, alemtuzumab continues to cause a decrease in CD4 counts for as long as 9 months after its discontinuation [29]. In the current study, alemtuzumab use in invasive aspergillosis was 6.5%, accounting for 30.8% of patients treated with alemtuzumab. Fludarabine was used in all cases of IFD. Although there are several reports that fludarabine, alemtuzumab, and ibrutinib increase the risk of PCP [30,31], there were no cases of PCP in patients using alemtuzumab in our study.

Our finding of no cancer drugs associated with PCP may be because we did not investigate whether PCP prophylaxis was administered. The effectiveness of preventive measures should be investigated in future studies. Ibrutinib, the first-line treatment for CLL, is a host risk factor that predisposes patients to fungal infections [10], especially the more common *Aspergillus*-related IFD [32]. Additionally, 56% of patients treated with ibrutinib develop any-grade infection [33]. However, the incidence of IFD in patients who received ibrutinib was the lowest among all other anticancer drugs evaluated in the present study. In contrast, the incidence of IFD in patients who received ibrutinib as first-line therapy was 2.9%, while the incidence of IFD in patients who received ibrutinib after second-line therapy was 12.3%. Ibrutinib use after third-line therapy has been reported to be a risk factor for IFD [34], similar to our results. Careful attention should be paid to the development of IFD when ibrutinib is used after second- or third-line therapy. The introduction of ibrutinib in 2016 has changed CLL treatment in Japan. The current study evaluated patients registered from 2009 to 2019, which may have resulted in the small number of patients using ibrutinib. Moreover, there were more cases of IFD among ibrutinib-treated patients (19/231 (8.2%)). Second-generation BTK inhibitors, such as acalabrutinib [35] and zanubrutinib [36], are now available; thus, the incidence of IFD may further increase in the future.

This study had some limitations. First, the definition of IFD cases in Japanese claims data has not been validated. To exclude patients without IFD as much as possible, we used both diagnostic codes and medications to define IFD. This methodology should be validated in future studies. Second, the claims data did not include certain clinical data, such as disease activity, laboratory data, and clinical course, which may influence the indication for IFD or the risk of IFD. Third, we have not evaluated the presence or absence of antifungal prophylaxis. Therefore, it was not possible to analyze the details of IFD, and the incidence may have been overestimated. In addition, we could not assess IFD risk according to cancer stage because we did not have information on the Rai and Binet classifications [9,37], which are necessary for CLL staging. Furthermore, the classification of IFD showed many unspecified IFDs because the data used did not include fungal cultures or polymerase chain reaction results. Fourth, we could not confirm previous treatment history at institutions not included in our database; thus, DT history may have been inaccurate. Other databases such as the national database and the Japan Medical Data Center database should be used to evaluate the incidence of IFD in patients with CLL considering DT history.

## 5. Conclusions

The incidence of IFD in Japan was significantly higher in the DT group than in the WW group, but there was no significant between-group difference in the time to IFD onset or type of IFD. To the best of our knowledge, this study is the first to determine the incidence of IFD in patients with CLL during WW. Physicians should monitor patients for IFD, even among those with CLL undergoing the WW protocol.

## Figures and Tables

**Table 1:** Baseline patient characteristics.

	Watch-and-Wait Group *n* = 2395	Drug Therapy Group *n* = 1089	*p*-Value
Age (years), median (IQR)	72 (64–81)	72 (64–79)	0.036
≥75 years, *n* (%)	1056 (44.1)	445 (40.9)	0.077
Male sex, *n* (%)	1341 (56.0)	693 (63.6)	<0.001
CCI score, median (IQR)	2 (2–3)	2 (2–3)	0.062
CCI score ≥ 5, *n* (%)	104 (4.3)	70 (6.4)	0.012
Comorbidities			
Diabetes mellitus, *n* (%)	728 (30.4)	347 (31.9)	0.384
Medication			
Steroid	289 (12.1)	349 (32.0)	<0.001
Anticancer drugs for the treatment of CLL			
Cyclophosphamide, *n* (%)	-	512 (47.0)	-
Rituximab, *n* (%)	-	490 (45.0)	-
Fludarabine, *n* (%)	-	428 (39.3)	-
Ibrutinib, *n* (%)	-	231 (21.2)	-
Bendamustine, *n* (%)	-	187 (17.2)	-
Ofatumumab, *n* (%)	-	43 (3.9)	-
Alemtuzumab, *n* (%)	-	13 (1.2)	-

Abbreviations: CLL, chronic lymphocytic leukemia; CCI, Charlson Comorbidity Index; IQR, interquartile range.

**Table 2:** Characteristics of invasive fungal diseases.

	Overall Group *n* = 135	Watch-and-Wait Group *n* = 35	Drug Therapy Group *n* = 100	*p*-Value
Invasive aspergillosis, *n* (%)	38 (28.1)	10 (28.6)	28 (28.0)	1
Invasive candidiasis, *n* (%)	9 (6.7)	0	9 (9.0)	0.111
Cryptococcosis, *n* (%)	2 (1.5)	1 (2.9)	1 (1.0)	0.453
Pneumocystis pneumonia, *n* (%)	20 (14.8)	5 (14.3)	15 (15.0)	1
Other IFD NOS, *n* (%)	29 (21.5)	8 (22.9)	21 (21.0)	0.814
Unspecified IFD, *n* (%)	37 (27.4)	11 (31.4)	26 (26.0)	0.519

IFD, Invasive fungal disease; NOS, not otherwise specified.

**Table 3:** Risk factors of invasive fungal diseases.

	Univariate		Multivariate	
	Hazard Ratio (95% CI)	*p*-Value	Hazard Ratio (95% CI)	*p*-Value
Age ≥ 75 years	0.928 (0.65–1.32)	0.677	1.03 (0.73–1.47)	0.855
Male sex	1.63 (1.13–2.34)	0.009	1.43 (0.99–2.06)	0.057
CCI score ≥ 5	1.60 (0.81–3.15)	0.175	1.24 (0.63–2.45)	0.537
Diabetes mellitus	1.25 (0.87–1.79)	0.232	1.20 (0.83–1.72)	0.334
Steroid	3.10 (2.20–4.36)	<0.001	2.13 (1.50–3.03)	<0.001
Drug therapy	5.15 (3.50–7.57)	<0.001	4.19 (2.82–6.23)	<0.001

CI, confidence interval; CCI, Charlson Comorbidity Index.

**Table 4:** Incidence of IFDs in patients treated with each treatment drug for CLL.

Treatment Drugs	Incidence (%)						
	All IFDs	IA	IC	Cryptococcosis	PCP	Other IFD NOS	Unspecified IFD
Cyclophosphamide	10.2	3.3	0.6	0	0.8	2.7	2.7
Rituximab	9.0	2.9	0.6	0	1.2	1.6	2.7
Fludarabine	12.4	3.0	1.6	0.2	1.9	3.0	2.6
Ibrutinib	8.2	2.2	0.9	0	1.7	1.3	2.2
Bendamustine	12.8	4.3	1.1	0	1.6	2.1	3.7
Ofatumumab	14.0	2.3	2.3	0	2.3	4.7	2.3
Alemtuzumab	38.5	30.8	0	0	0	0	7.7

IFD; invasive fungal disease; IA, invasive aspergillosis; IC, invasive candidiasis; PCP, pneumocystis pneumonia; NOS, not otherwise specified.

## Data Availability

The data are not publicly available as the participants of this study did not agree for their data to be shared publicly.
